# Supplementary material for: Long-Term Immunogenicity and Efficacy of the Oral Rabies Virus Vaccine Strain SPBN GASGAS in Foxes
Source: Viruses. 2019 Aug 27;11(9):790. doi: 10.3390/v11090790 (PMC6784248; doi:10.3390/v11090790)
Supplement: Supplementary file 1 [file viruses-11-00790-s001.pdf]

			B0		B1		B2		B3		B4		B5		B6		B7		B8		B9		post mortem		B10	
			-1 week		3 weeks p.v.		5 weeks p.v.		9 weeks p.v.		18 weeks p.v.		26 weeks p.v.		39 weeks p.v.		53 weeks p.v.		55 weeks p.v.		57 weeks p.v.		post mortem		66 weeks p.v.	
**Group**	**Status**	**Animal ID**	**RFFIT IU/ml**	**ELISA % inhibition**	**RFFIT IU/ml**	**ELISA % inhibition**	**RFFIT IU/ml**	**ELISA % inhibition**	**RFFIT IU/ml**	**ELISA % inhibition**	**RFFIT IU/ml**	**ELISA % inhibition**	**RFFIT IU/ml**	**ELISA % inhibition**	**RFFIT IU/ml**	**ELISA % inhibition**	**RFFIT IU/ml**	**ELISA % inhibition**	**RFFIT IU/ml**	**ELISA % inhibition**	**RFFIT IU/ml**	**ELISA % inhibition**	**RFFIT IU/ml**	**ELISA % inhibition**	**RFFIT IU/ml**	**ELISA % inhibition**
		Fu2	0,27	3,95	1,16	82,63	1,16	91,89	2,20	96,03	1,69	98,48	3,73	100,22	5,18	99,39	1,20	96,51	5,02	98,81	8,27	98,28			2,38	93,15
		Fu3	0,07	12,55	0,09	67,29	0,23	74,78	0,31	87,01	0,98	83,10	0,77	84,75	0,80	83,26	0,37	69,27	1,45	94,15	8,16	95,99			1,73	92,01
		Fu6	0,07	12,62	4,05	83,90	1,49	95,30	5,05	97,34	0,42	100,71	4,43	100,29	8,97	100,78	1,95	94,24	33,27	100,66	101,57	100,53			9,47	99,18
		Fu7	0,11	16,01	3,56	81,53	1,69	91,08	1,22	90,77	1,57	98,65	3,45	95,72	6,25	97,66	1,19	91,49	24,32	100,30	41,72	100,51			5,04	97,34
		Fu9	0,07	11,34	1,69	91,92	2,05	94,32	1,56	98,41	5,38	100,90	2,31	101,25	3,39	101,15	2,26	99,78	22,17	100,39	43,90	100,50			8,98	99,37
		Fu11	0,14	14,36	2,47	82,91	1,49	92,88	1,38	99,54	0,94	99,84	0,68	101,26	0,22	95,70	1,06	98,82	6,11	98,62	30,23	100,41			5,97	99,41
		Fu12	0,06	15,84	16,95	85,71	5,45	96,78	4,72	99,25	1,65	101,06	6,11	101,34	13,39	97,18	8,27	100,22	43,59	99,79	81,87	99,95			14,45	98,49
		Fu13	0,12	4,21	1,95	83,74	0,81	91,25	1,13	95,92	1,61	100,16	3,21	100,16	1,08	96,15	1,43	95,26	43,73	78,52	69,08	99,56			17,05	99,53
		Fu14	0,13	21,23	0,09	41,66	0,16	75,89	0,26	79,15	0,10	64,81	0,40	77,03	0,02	52,47	0,04	39,96	15,79	94,33	27,87	94,43			0,82	82,76
		Fu15	0,15	14,59	0,05	20,59	0,07	21,43	0,29	12,01	0,02	24,82	0,03	19,43	0,06	-7,54	0,04	10,96	0,63	48,33			15,55	72,80		
		Fu16	0,37	12,26	2,18	62,17	1,35	86,92	1,26	92,62	1,19	98,16	1,50	98,71	1,40	93,47	0,92	95,11	33,24	100,70	12,91	99,49			2,17	98,87
		Fu19	0,01	9,68	1,88	83,50	1,20	90,05	2,70	96,18	3,24	99,20	4,12	101,14	1,97	96,63	1,67	98,15	69,80	98,27	18,19	96,75			18,37	99,18
		Fu20	0,03	11,71	7,96	86,22	3,80	90,96	1,68	94,94	4,23	97,98	1,74	98,38	1,10	91,21	1,10	84,33	125,11	100,37	27,57	99,75			22,58	99,32
		Fu21	0,11	16,17	1,02	81,27	0,53	89,05	0,84	97,10	3,10	100,55	1,57	100,93	2,04	98,25	2,33	96,20	43,72	99,84	7,84	98,98			3,61	98,43
		Fu24	0,10	12,99	0,63	66,05	0,40	83,81	0,93	91,88	0,95	95,23	1,03	93,79	1,09	87,37	0,35	87,81	19,52	98,04	6,65	96,26			3,60	93,67
Group 1	vaccinated	Fu26	0,04	-0,68	10,94	91,22	2,55	93,53	4,32	99,30	13,08	101,16	10,09	101,21	6,20	97,53	3,47	99,90	50,24	100,49	15,29	99,21			20,72	100,03
		Fu27	0,11	19,65	0,20	71,35	0,50	82,16	0,86	93,36	2,22	98,73	3,29	99,35	3,70	95,25	2,31	96,00	32,98	99,66	10,93	99,46			4,24	98,55
		Fu28	0,03	13,09	1,60	86,34	1,08	91,05	3,39	99,77	4,87	100,95	8,89	101,05	9,82	98,96	3,73	98,61	59,04	98,92	19,29	99,38			12,05	100,26
		Fu30	0,04	18,72	19,55	93,23	9,36	96,86	12,99	97,63	10,69	100,68	9,81	100,79	10,27	98,66	35,65	100,14	40,11	102,09	43,40	101,86			15,23	99,68
		Fu31	0,06	22,05	8,28	91,21	5,83	97,20	44,20	98,86	24,59	101,61	43,83	101,35	24,80	99,42	36,36	100,78	63,69	103,01	50,66	102,49			18,41	100,15
		Fu32	0,07	21,51	4,65	87,88	8,16	96,18	23,39	98,26	27,55	99,96	101,87	98,92												
		Fu33	0,02	18,46	8,97	85,65	8,48	94,73	30,84	98,54	24,07	100,17	18,57	101,25	21,50	99,23	24,40	99,37	64,96	98,36	50,66	100,46			43,78	100,60
		Fu34	0,04	15,22	0,02	14,70	0,06	10,83	0,12	9,92	0,06	22,15	0,08	22,80	0,07	-4,31	0,03	-4,30					49,80	73,63		
		Fu35	0,03	17,92	1,71	82,03	1,27	86,33	2,13	93,97	1,72	97,43	2,47	96,41	2,69	96,79	2,41	97,14	64,96	100,15	32,25	100,83			13,72	100,68
		Fu38	0,13	18,19	1,97	81,61	0,81	85,54	3,41	94,83	1,22	98,93	3,45	97,97	2,03	95,41	2,73	96,42	53,69	100,66	14,39	100,70			3,28	99,51
		Fu40	0,21	19,71	1,03	89,11																				
		Fu41	0,02	18,46	0,01	0,11	0,01	8,31	0,05	12,92	0,04	9,97	0,03	20,81	0,06	-5,40	0,07	-2,83					3,34	70,19		
		Fu42	0,06	16,75	26,56	78,26	8,40	92,19	9,47	96,82	15,83	98,96	6,56	101,76	8,51	101,36	12,85	98,74								
		Fu43	0,03	12,35	0,76	84,81	1,37	93,68	3,33	97,76	5,02	100,04	7,40	95,07	3,19	99,82	2,20	73,72	47,72	99,69	24,13	102,29			7,12	99,44
		Fu44	0,25	13,43	0,84	68,97	0,22	83,25	0,32	90,35	0,42	94,18	0,35	101,35	0,35	96,31	0,27	93,33	73,14	99,86	34,43	103,31			6,55	99,87
		Fu45	0,06	21,51	9,47	88,31	12,25	95,56	5,58	98,45	10,27	100,50	21,42	100,37	18,76	98,67	7,43	99,92	8,76	98,61	4,05	99,92			0,43	93,61
		Fu1	0,16	12,26	0,02	23,25	0,09	16,28	0,06	7,79	0,02	17,47	0,03	23,85	0,04	21,09	0,10	20,84	0,93	57,67			2,68	68,31		
		Fu4	0,09	11,19	0,10	28,67	0,07	22,50	0,17	12,42	0,01	21,25	0,08	25,76	0,12	16,12	0,03	6,47	0,14	18,48			6,98	55,65		
		Fu5	0,09	14,94	0,04	9,80	0,08	17,87	0,14	10,44	0,04	26,04	0,18	21,62	0,12	22,25	0,05	2,15	2,53	53,64			7,48	66,30		
		Fu8	0,24	11,55	0,27	16,22	0,64	15,86	0,54	8,87	0,08	25,27	0,53	24,87	0,04	17,34	0,03	11,84	6,26	64,39			52,03	75,02		
		Fu10	0,07	3,43	0,40	19,93	0,18	12,09	0,09	10,40	0,08	18,01	0,05	25,45	0,17	-7,82	0,04	11,18	0,09	28,68			4,81	45,05		
		Fu17	0,10	14,14	0,10	18,06	0,04	15,83	0,12	14,39	0,04	24,34	0,02	27,42	0,05	-6,31	0,07	15,29	10,01	65,40	8,64	90,03			5,71	98,49
		Fu18	0,09	4,17	0,08	21,62	0,07	15,46	0,74	17,98	0,50	12,15	0,05	27,89	0,34	-12,57	0,10	8,78	37,02	54,38			38,01	72,02		
Group 2	control	Fu22	0,09	12,90	0,10	12,56	0,09	16,04	0,12	13,53	0,03	23,73	0,19	22,37	0,07	10,20	0,01	-11,90	14,16	66,16						
		Fu23	0,04	17,21	0,12	24,73	0,11	20,21	0,27	12,69	0,06	22,32	0,32	19,70	0,06	15,05										
		Fu25	0,11	11,16	0,06	23,75	0,12	17,19	0,39	16,99	0,05	23,14	0,28	24,59	0,15	-2,24	0,05	49,36	109,47	56,47			44,11	78,68		
		Fu29	0,08	13,61	0,03	9,68	0,07	11,14	0,14	6,66	0,02	19,51	0,06	13,74	0,09	-15,07	0,06	-9,24					6,32	71,80		
		Fu36	0,05	14,86	0,06	20,03	0,09	10,54	0,02	3,63	0,02	16,45	0,07	14,34	0,08	-15,96	0,07	-5,58					57,67	64,87		
		Fu37	0,09	11,54	0,24	11,78	0,10	3,84	0,08	-0,84	0,02	18,95	0,17	10,82	0,10	-7,42	0,14	-9,88					62,20	80,81		
		Fu39	0,07	20,16	0,10	15,01	0,25	21,11	0,13	22,46	0,02	23,37	0,07	18,64	0,09	7,20	0,08	2,55					627,46	74,57		
		Fu46	0,13	23,04	0,02	9,35	0,11	15,25	0,04	32,12	0,04	21,71	0,02	13,21	0,09	2,06	0,01	-4,36	23,04	99,89	12,53	102,55	3,22	56,79	4,58	100,60
